# Equine major histocompatibility complex class I molecules act as entry receptors that bind to equine herpesvirus-1 glycoprotein D

**DOI:** 10.1111/j.1365-2443.2011.01491.x

**Published:** 2011-04

**Authors:** Michihito Sasaki, Rie Hasebe, Yoshinori Makino, Tadaki Suzuki, Hideto Fukushi, Minoru Okamoto, Kazuya Matsuda, Hiroyuki Taniyama, Hirofumi Sawa, Takashi Kimura

**Affiliations:** 1 Department of Molecular Pathobiology, Hokkaido University Research Center for Zoonosis ControlSapporo 001-0020, Japan; 2 Global COE Program, Hokkaido UniversitySapporo 060-0818, Japan; 3 Department of Applied Veterinary Sciences, United Graduate School of Veterinary Sciences, Gifu UniversityGifu 501-1193, Japan; 4 Laboratory of Veterinary Microbiology, Faculty of Applied Biological Sciences, Gifu UniversityGifu 501-1193, Japan; 5 Department of Veterinary Pathology, School of Veterinary Medicine, Rakuno Gakuen UniversityEbetsu, Hokkaido 069-8501, Japan

## Abstract

The endotheliotropism of equine herpesvirus-1 (EHV-1) leads to encephalomyelitis secondary to vasculitis and thrombosis in the infected horse central nervous system (CNS). To identify the host factors involved in EHV-1 infection of CNS endothelial cells, we performed functional cloning using an equine brain microvascular endothelial cell cDNA library. Exogenous expression of equine major histocompatibility complex (MHC) class I heavy chain genes conferred susceptibility to EHV-1 infection in mouse NIH3T3 cells, which are not naturally susceptible to EHV-1 infection. Equine MHC class I molecules bound to EHV-1 glycoprotein D (gD), and both anti-gD antibodies and a soluble form of gD blocked viral entry into NIH3T3 cells stably expressing the equine MHC class I heavy chain gene (3T3-A68 cells). Treatment with an anti-equine MHC class I monoclonal antibody blocked EHV-1 entry into 3T3-A68 cells, equine dermis (E. Derm) cells and equine brain microvascular endothelial cells. In addition, inhibition of cell surface expression of MHC class I molecules in E. Derm cells drastically reduced their susceptibility to EHV-1 infection. These results suggest that equine MHC class I is a functional gD receptor that plays a pivotal role in EHV-1 entry into equine cells.

## Introduction

Equine herpesvirus-1 (EHV-1), an alphaherpesvirus of the family *Herpesviridae* with a worldwide distribution, can cause respiratory disease, abortion and encephalomyelitis in horses. Although encephalomyelitis is uncommon, outbreaks of neurologic EHV-1 have caused great damage to the equine industry (Stierstorfer *et al.* 2002; Studdert *et al.* 2003; Kohn *et al.* 2006; Heerkens 2009); still, neither vaccination nor effective treatments are available for this disease. Following airborne transmission, EHV-1 infects respiratory epithelial cells and mononuclear leukocytes in the local lymph nodes, resulting in leukocyte-associated viremia. The virus then infects endothelial cells of arteries and capillaries in the central nervous system (CNS). Previous research has shown that the inflammation following viral replication in the endothelial cells triggers encephalomyelitis secondary to vasculitis, thrombosis and ischemic damage of the CNS (Edington *et al.* 1986; Whitwell & Blunden 1992; Stierstorfer *et al.* 2002). However, the mechanisms underlying EHV-1 endotheliotropism still need to be elucidated.

Alphaherpesviruses, including herpes simplex virus type 1 (HSV-1), HSV-2, varicella-zoster virus (VZV) and pseudorabies virus (PRV), enter target cells through a sequential multistep process. Following the initial attachment of the viruses to the cell surface, binding of viral glycoproteins to cell surface receptors triggers fusion of the viral envelope with the cell membrane, resulting in the release of viral capsid (containing viral genome) into the cytoplasm. Various alphaherpesvirus receptors have been previously identified, including a member of the tumor necrosis factor receptor family referred to as herpesvirus entry mediator (HVEM or HveA); the members of the immunoglobulin superfamily nectin-1 (HveC), nectin-2 (HveB) and CD155 (HveD); 3-*O*-sulfated heparan sulfate; paired immunoglobulin-like type 2 receptor α; nonmuscle myosin IIA; insulin-degrading enzyme and myelin-associated glycoprotein (Montgomery *et al.* 1996; Geraghty *et al.* 1998; Warner *et al.* 1998; Shukla *et al.* 1999; Li *et al.* 2006; Satoh *et al.* 2008; Arii *et al.* 2010; Suenaga *et al.* 2010). Chinese hamster ovary (CHO)-K1 cells are naturally resistant to HSV-1, HSV-2, PRV and VZV infection, but these viruses can infect CHO-K1 cells transfected with the corresponding receptor. In contrast, EHV-1 efficiently enters and replicates in CHO-K1 cells, suggesting that EHV-1 utilizes a unique entry receptor (Frampton *et al.* 2005).

EHV-1 attaches to cell surfaces using an interaction between viral glycoprotein C (gC) and cell surface heparan sulfate (Osterrieder 1999). Although the role of gC is important for effective infection, it does not trigger viral entry into cells. Entry of EHV-1 occurs either by endocytosis or by direct membrane fusion with the cell surface, depending on cell types and possibly on viral strains (reviewed in Osterrieder & Van de Walle 2010). Glycoprotein D (gD) of EHV-1 is known to be essential for EHV-1 entry into rabbit kidney (RK13) and CHO-K1 cells (Frampton *et al.* 2005; Whalley *et al.* 2007). It has been shown that αV integrin mediates entry of the EHV-1 strain L11Δgp2 into both CHO-K1 cells and equine peripheral blood mononuclear cells (PBMC) through the interaction with gD, but does not facilitate EHV-1 entry into equine vascular endothelial cells (Van de Walle *et al.* 2008). The functional gD receptors that mediate EHV-1 entry into equine vascular endothelial cells remain uncertain.

Primary cultured equine brain microvascular endothelial cells (EBMECs) are an appropriate *in vitro* model for EHV-1 endotheliotropism studies (Hasebe *et al.* 2006). In this paper, we identified an equine major histocompatibility complex (MHC) class I heavy chain gene that rendered NIH3T3 cells susceptible to EHV-1 infection, from a cDNA library of primary cultured EBMECs. Equine MHC class I directly interacted with EHV-1 gD, a viral protein known to be important for EHV-1 entry. Interestingly, EHV-1 dependence on MHC class I for entry was observed in equine cell types, but not in CHO-K1, which is a nonequine cell line also susceptible to EHV-1 infection. Collectively, these results suggest that equine MHC class I acts as a gD receptor for EHV-1 entry into equine cell types, including CNS endothelial cells.

## Results

### Complementary DNA library screening

**Figure 1:**
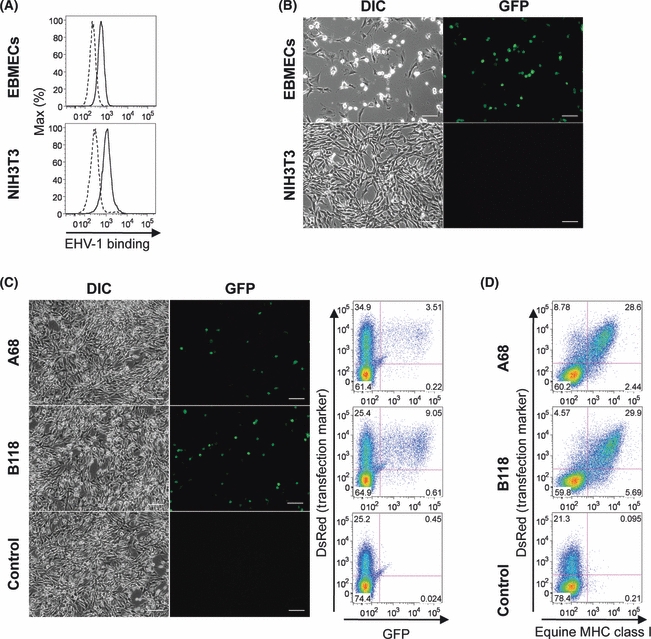
Equine herpesvirus-1 (EHV-1) infection of NIH3T3 cells expressing equine major histocompatibility complex (MHC) class I. (A) Flow cytometric detection of EHV-1 virions bound to the cell surface of equine brain microvascular endothelial cells (EBMECs) (upper panel) and NIH3T3 cells (lower panel). EHV-1-treated (solid line) and mock-treated (dashed line) cells were stained with an anti-EHV-1 antibody. (B) EBMECs and NIH3T3 cells were inoculated with Ab4-GFP at an MOI of 5 for 12 h. EHV-1 infected cells were identified by observing green fluorescent protein (GFP) signals under a fluorescence microscope. Scale bars: 100 μm. (C) NIH3T3 cells transiently transfected with pA68-DsRed, pB118-DsRed or pluc-DsRed were infected with EHV-1 Ab4-GFP at an MOI of 5. The number of GFP- and DsRed-positive cells was analyzed simultaneously by flow cytometry. Scale bars: 100 μm. (D) The expression of equine MHC class I was analyzed by flow cytometry using an anti-equine MHC class I antibody PT85A. Numbers on plots indicate the percentage of cells in the designated gate.

We first examined the attachment of EHV-1 to EBMECs, which are highly susceptible to EHV-1 infection, and to murine fibroblast-derived NIH3T3 cells, which are considered resistant to EHV-1 infection. Viral attachment was detected in both EBMECs and NIH3T3 cells by flow cytometry (Fig. 1A). However, following infection of the cells with the green fluorescent protein (GFP)-expressing EHV-1 mutant strain (Ab4-GFP), the GFP signal was observed in EBMECs, but not in NIH3T3 (Fig. 1B). These results suggest that NIH3T3 cells are resistant to EHV-1 infection despite efficient cell surface viral attachment.

We next screened an equine brain microvascular endothelial cell cDNA library for sequences that rendered NIH3T3 cells susceptible to EHV-1 infection. In the first round of screening, plasmids from one group of 20 bacterial stocks from the cDNA library made six cells in the monolayer susceptible to Ab4-GFP infection. GFP-positive cells were entirely absent in the monolayer transfected with mock plasmids. To increase the frequency of conversion, we subdivided the bacterial stock showing the highest conversion frequency into groups of 10 and then repeated plasmid DNA transfection and screening. After five rounds of division and screening, we obtained the desired plasmid clone (pcDNA201-81-13-71-68). DNA sequencing of pcDNA201-81-13-71-68 revealed that a cDNA insert of 1587 bp encoded an open reading frame (ORF) of 365 amino acids (aa). A search of the GenBank/EMBL/DDBJ databases showed that this protein product exhibited a similarity of 91% with the equine MHC class I heavy chain *ECMHCA1* (GenBank/EMBL/DDBJ entry X71809).

According to the classification of equine MHC class I genes reported by Holmes & Ellis (1999), the deduced aa sequence belonged to group A of the class I sequence; therefore, we designated the cloned cDNA (GenBank/EMBL/DDBJ entry AB525079) as A68. A68 was subcloned from pcDNA201-81-13-71-68 into the expression plasmid, pIRES2-DsRed-Express2, and the resulting plasmid was designated as pA68-DsRed. After inoculation of Ab4-GFP, NIH3T3 cells transfected with pA68-DsRed displayed cytopathic effects (CPE) and GFP expression, whereas NIH3T3 cells transfected with control plasmid, pluc-DsRed encoding luciferase, showed neither CPE nor GFP expression (Fig. 1C). Nearly all GFP-positive cells showed DsRed positivity by flow cytometry analysis, suggesting that EHV-1 infection occurred only in NIH3T3 cells successfully transfected with pA68-DsRed (Fig. 1C).

Because A68 was assigned to the MHC class I gene family, we evaluated whether other equine MHC class I heavy chain genes enhanced susceptibility to EHV-1 infection. A cDNA encoding equine MHC class I heavy chain that was 100% identical to the *Equine classical MHC class I allele 118 partial cds* (GenBank/EMBL/DDBJ entry AY176106) was isolated from EHV-1-susceptible equine dermal fibroblast (E. Derm) cells by reverse transcriptase PCR (RT-PCR). Because the deduced aa sequence of the isolated equine MHC class I heavy chain belonged to group B of the MHC sequence (Holmes & Ellis 1999), we designated the cloned cDNA (GenBank/EMBL/DDBJ entry AB525080) as B118 and constructed pB118-DsRed by cloning it into the pIRES2-DsRed-Express2 expression vector. NIH3T3 cells transfected with pB118-DsRed also showed CPE and GFP expression after inoculation with Ab4-GFP (Fig. 1C). Flow cytometry using the anti-MHC class I antibody PT85A, a monoclonal antibody that recognizes horse MHC class I but not mouse MHC class I, confirmed the cell surface expression of equine MHC class I in NIH3T3 cells transfected with pA68-DsRed or pB118-DsRed, but not in those transfected with control plasmid (Fig. 1D). We also isolated mouse MHC class I, H2K, and H2D from mouse spleen cDNA and confirmed that the over-expression of murine MHC class I did not confer susceptibility to EHV-1 infection (data not shown). These results indicate that cell surface expression of equine MHC class I renders NIH3T3 cells susceptible to EHV-1 infection.

### Effect of equine MHC class I expression on NIH3T3 cells

**Figure 2:**
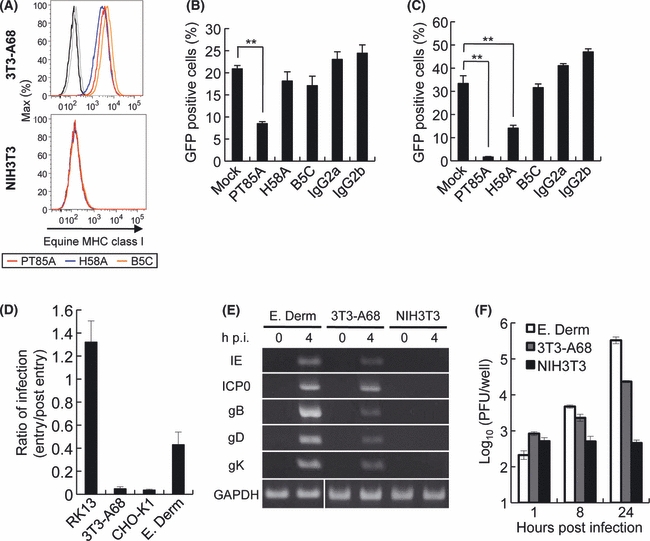
Effect of equine major histocompatibility complex (MHC) class I expression on NIH3T3 cells. (A) Flow cytometric detection of equine MHC class I expression on 3T3-A68 (upper panel) and NIH3T3 cells (lower panel) stained with anti-MHC class I antibody PT85A (red), H58A (blue), B5C (orange) or isotype controls IgG2a and IgG2b (black and gray, respectively). (B and C) Infectivity neutralizing assay. 3T3-A68 cells (B) and equine brain microvascular endothelial cells (EBMECs) (C) were preincubated with 50 μg/mL of anti-MHC class I or control antibodies for 30 min and then infected with equine herpesvirus-1 (EHV-1) Ab4-GFP at an MOI of 5 in the presence of the antibody for 2 h at 37 °C. After treatment with a low-pH citrate buffer to inactivate extracellular virions, incubation was continued for a further 12 h. Green fluorescent protein (GFP)-positive cells were counted by flow cytometry. Bars represent the means from three samples, and error bars show standard deviations. Statistical significance was determined by Student's *t*-tests and is indicated by asterisks (**P < 0.01). (D) Effect of cellular ATP depletion on EHV-1 entry into cells. RK13, 3T3-A68, CHO-K1 and E. Derm cells were treated with ATP depletion media during or postviral entry. These cells were infected with Ab4-GFP, and GFP-positive cells were counted by flow cytometry. The number of infected cells treated with ATP depletion media postviral entry was defined as one. The graphs show the mean and standard deviation of three independent experiments. (E) Expression of viral RNAs in 3T3-A68, NIH3T3 and E. Derm cells. Cells were infected with EHV-1 Ab4-GFP, and total RNA was extracted at 0 and 4 h postinfection (p.i.) Immediate early (IE), early (ICP0) and late (gB, gD, gK) transcripts were detected by RT-PCR. GAPDH was used as an internal control. (F) Time course of viral growth in E. Derm (white bars), 3T3-A68 (gray bars) and NIH3T3 (black bars) cells. Cells were infected to Ab4-GFP at an MOI of 5 for 1h and washed with PBS to remove uninfected virus. The viral titer of each supernatant was determined by a plaque formation assay on RK13 cells. Each bar represents the amount of cell-free virus as the mean of three independent samples. Error bars show standard deviations.

To further analyze the function of equine MHC class I molecules in EHV-1 infection, we established an NIH3T3-derived cell line that stably expresses A68 (3T3-A68). Anti-equine MHC class I monoclonal antibodies PT85A (Isotype IgG2a), H58A (Isotype IgG2a) and B5C (Isotype IgG2b) specifically reacted with A68 on the cell surface of 3T3-A68 (Fig. 2A). We attempted to neutralize viral infection by incubating cells with these anti-MHC class I antibodies. After preincubation with the antibodies or the IgG isotype controls, cells were exposed to Ab4-GFP. Viral entry was evaluated by counting the number of GFP-expressing cells using flow cytometry. PT85A treatment significantly inhibited virus entry into 3T3-A68 (inhibition of 59% compared to the mock treatment), but H58A and B5C did not block viral entry significantly (Fig. 2B). We also investigated the neutralizing activity of anti-MHC class I antibodies on EBMECs. Expression of MHC class I on EBMECs was confirmed by flow cytometry (Fig. S1 in Supporting Information). PT85A and H58A prevented EHV-1 entry into EBMECs (infection inhibition of 95% and 62%, respectively) (Fig. 2C). These results suggest that equine MHC class I molecules are involved in EHV-1 entry into both 3T3-A68 cells and EBMECs.

We next examined the effects of cellular ATP depletion on EHV-1 entry into 3T3-A68 cells. ATP depletion is known to inhibit endocytosis, but has no effect on herpesvirus entry by direct fusion of viral envelopes with plasma membranes (Nicola *et al.* 2003). Previous studies have suggested that entry of EHV-1 into CHO-K1 cells occurs via an endocytic mechanism (Frampton *et al.* 2007; Van de Walle *et al.* 2008), whereas entry into RK13 cells occurs by direct fusion at the cell surface (Frampton *et al.* 2007). Therefore, we used CHO-K1 and RK13 cells as positive and negative controls, respectively, for determining the effect of cellular ATP depletion on EHV-1 entry. We also used E. Derm cells as a positive control because EHV-1 entry into E. Derm cells was reduced by ATP depletion (Hasebe *et al.* 2009). An ATP depletion experiment was performed by using the method described by Hasebe *et al.* (2009) with minor modifications. To exclude the toxic influence of ATP depletion on cell viability, the effect of ATP depletion on EHV-1 entry was estimated by dividing the number of infected cells obtained from the ATP depletion media during viral infection (viral entry step) by the number of infected cells obtained from the ATP depletion media after viral infection (postviral entry step). ATP depletion during viral entry reduced EHV-1 infection in 3T3-A68 as well as CHO-K1 and E. Derm cells (Fig. 2D). In contrast, EHV-1 infection was not inhibited in RK13 cells (Fig. 2D). The observed decrease in cellular ATP levels in each cell line (Fig. S2 in Supporting Information) suggests that EHV-1 entry into 3T3-A68 cells depends on cellular ATP, which is required for viral cell entry via endocytosis.

We performed RT-PCR to confirm EHV-1 gene expression in 3T3-A68 cells using specific primer sets for immediate early (IE), early (ICP0) and late (gB, gD and gK) EHV-1 genes. At 4 h postinfection (p.i.), all genes were detected in 3T3-A68 and E. Derm cells, whereas no viral RNA was detected in NIH3T3 cells (Fig. 2E). Because no PCR product was detected in the samples without transcriptase, amplified products were not derived from the viral genomic DNA (data not shown). We also investigated whether 3T3-A68 cells could support EHV-1 replication by evaluating the kinetics of viral growth. The extracellular titers of 3T3-A68 and E. Derm cells gradually increased with time (Fig. 2F). Conversely, EHV-1-inoculated NIH3T3 cells yielded no infectious progeny at 24 h p.i. (Fig. 2F). These results suggest that equine MHC class I molecules mediate EHV-1 entry and subsequent replication in 3T3-A68 cells.

### Involvement of EHV-1 gD on equine MHC class I-mediated viral entry

**Figure 3:**
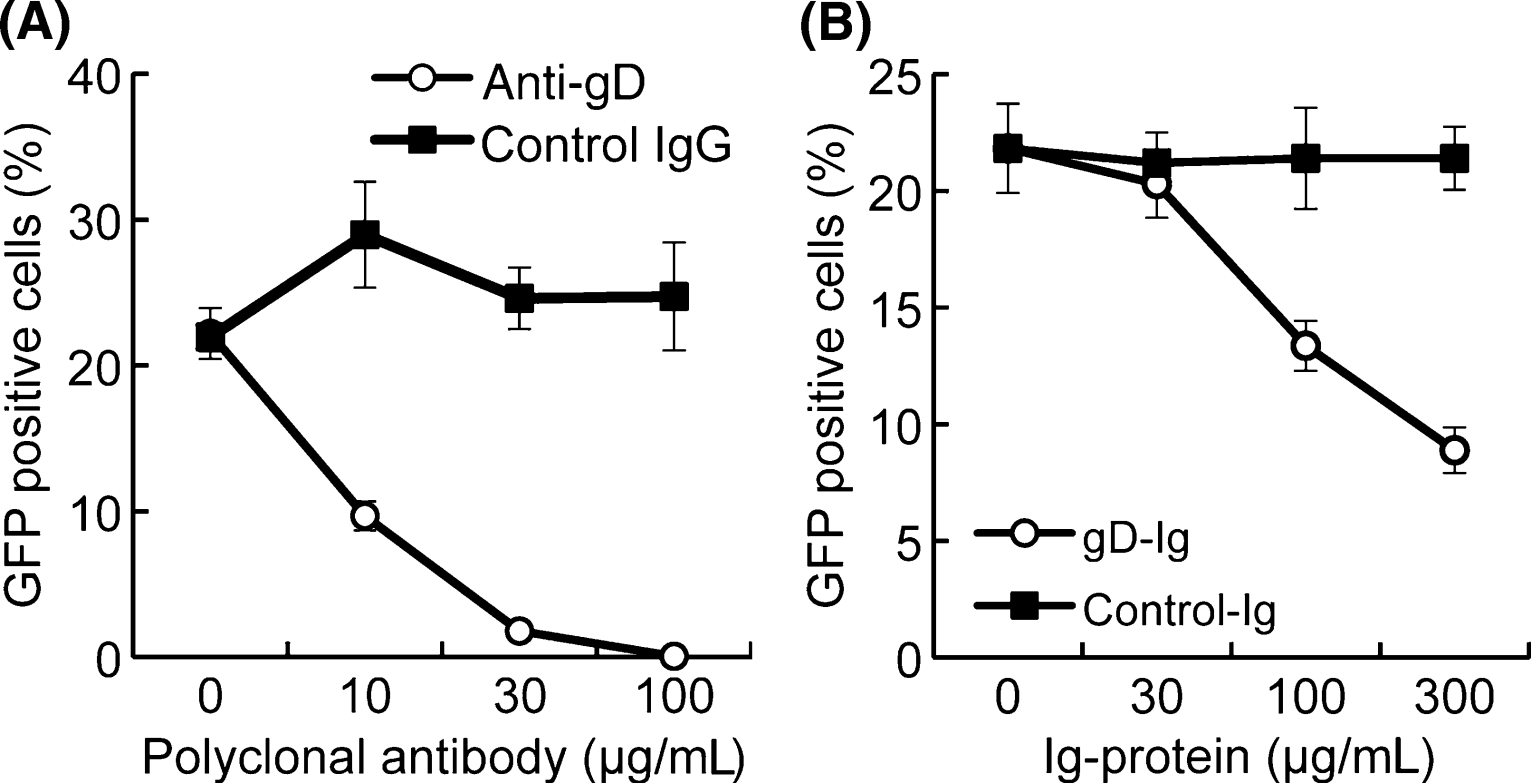
Involvement of equine herpesvirus-1 (EHV-1) glycoprotein D (gD) in equine major histocompatibility complex (MHC) class I-mediated viral entry. (A) Inhibition of EHV-1 entry by anti-gD polyclonal antibody. EHV-1 Ab4-GFP at a final MOI of 5 was incubated with anti-gD polyclonal antibody (open circles) or control rabbit IgG (solid squares) for 30 min, and the virus–antibody mixture was added to 3T3-A68 cells. After incubation for 2 h, extracellular virus was inactivated by treatment with citrate buffer. Green fluorescent protein (GFP)-positive cells were counted by flow cytometry. (B) Inhibition of EHV-1 entry by gD-Ig fusion protein. 3T3-A68 cells were incubated with gD-Ig (open circles) or control-Ig (solid squares) for 30 min and infected with the EHV-1 Ab4-GFP at an MOI of 5. After incubation for 2 h, extracellular virus was inactivated by treatment with citrate buffer. GFP-positive cells were counted by flow cytometry. Error bars represent standard deviations of three independent samples.

Glycoprotein D (gD) of EHV-1 is known to be important for EHV-1 entry into RK13 (Whalley *et al.* 2007) and CHO-K1 cells (Van de Walle *et al.* 2008). To evaluate the role of gD on equine MHC class I-mediated entry, we tested the ability of anti-gD polyclonal antibody to block EHV-1 infection of 3T3-A68 cells. Anti-gD polyclonal antibody inhibited EHV-1 entry in a dose-dependent manner (Fig. 3A). We next generated a soluble gD-Ig fusion protein consisting of the extracellular domain of EHV-1 gD and the Fc segment of the human immunoglobulin G1 (IgG1) and tested its ability to block EHV-1 infection of 3T3-A68 cells as previously described by Satoh *et al.* (2008). gD-Ig also inhibited EHV-1 entry into 3T3-A68 cells in a dose-dependent manner, with concentrations higher than 30 ug/mL being effective at blocking infection (Fig. 3B). Anti-gD antibody and gD-Ig also inhibited EHV-1 entry into 3T3-B118 cells, a NIH3T3-derived cell line stably expressing the other allele of equine MHC class I (data not shown). These results suggest that EHV-1 gD is involved in equine MHC class I-mediated EHV-1 entry.

**Figure 4:**
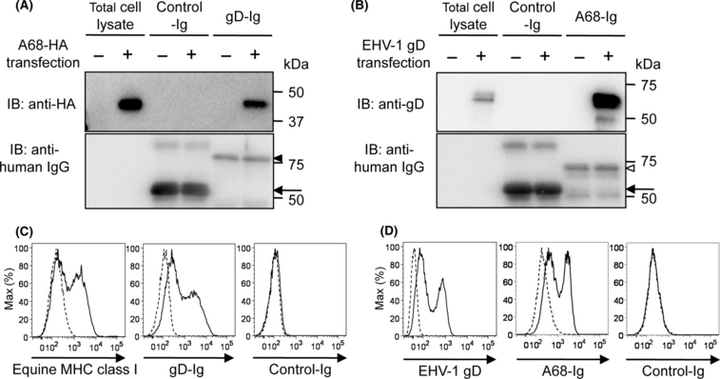
Interaction of equine major histocompatibility complex (MHC) class I with equine herpesvirus-1 (EHV-1) glycoprotein D (gD). (A, B) Immunoprecipitation assay. (A) Protein A beads coupled with gD-Ig or control-Ig were incubated with a lysate of 293T cells co-transfected with HA-tagged A68 and equine β2m expression plasmids. (B) Protein A beads coupled with A68-Ig or control-Ig were incubated with a lysate of 293T cells transfected with the EHV-1 gD expression plasmid. Precipitated proteins were separated by SDS-PAGE and subjected to immunoblotting (IB) using the antibodies indicated. Solid arrowhead, open arrowhead and arrow indicate gD-Ig, A68-Ig and control Ig, respectively. (C, D) Binding assay of gD and A68. (C) A68 and equine β2m co-transfected 293T cells (solid line) or mock vector transfected 293T cells (dashed lines) were incubated with anti-equine MHC class I monoclonal antibody (left), gD-Ig (middle) or control-Ig (right). (D) 293T cells transfected with EHV-1 gD (dashed lines) or mock vector (dashed lines) were incubated with anti-gD polyclonal antibody (left), A68-Ig (middle) or control-Ig (right). Bound Ig fusion proteins were stained with phycoerythrin-conjugated anti-human IgG and analyzed by flow cytometry.

To investigate the putative interaction between equine MHC class I and EHV-1 gD, lysates of cells expressing hemagglutinin (HA)-tagged A68 (A68-HA) were incubated with gD-Ig fusion protein using coupled magnetic beads. A68 was precipitated by gD-Ig but not by the control-Ig (Fig. 4A). We also confirmed that EHV-1 gD was precipitated by the soluble A68-Ig fusion protein (Fig. 4B). Next, we analyzed the direct binding of gD to A68 expressed on the cell surface, which induced binding of gD-Ig, but not control-Ig, to cells (Fig. 4C). Similarly, A68-Ig reacted with EHV-1 gD expressed on the cell surface (Fig. 4D). Cell surface expression of A68 and EHV-1 gD was confirmed by staining with specific antibodies (Fig. 4C,D). These results indicate that EHV-1 gD acts as a ligand for equine MHC class I molecules on the cell surface.

### Anti-MHC class I antibodies block EHV-1 entry into equine cells

**Figure 5:**
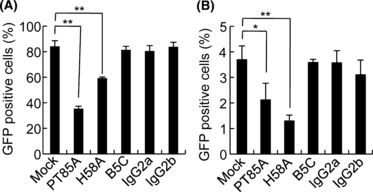
Inhibition of equine herpesvirus-1 (EHV-1) entry into equine cells with an anti-major histocompatibility complex (MHC) class I antibody. (A) E. Derm and (B) equine peripheral blood mononuclear cells were preincubated with 50 μg/mL of anti-MHC class I or control antibodies for 30 min and then infected with EHV-1 Ab4-GFP at an MOI of 5 in the presence of the antibody for 2 h at 37 °C. After removing virus–antibody mixtures, cells were treated with a low-pH citrate buffer to inactivate extracellular virions. Incubation was continued for a further 12 h. Green fluorescent protein (GFP)-positive cells were counted by flow cytometry. Bars represent the means from three samples, and error bars show standard deviations. Statistical significance was determined by Student's *t*-tests and is indicated by asterisks (*P < 0.05, **P < 0.01).

We investigated the involvement of MHC class I molecules in EHV-1 entry into other equine cell types that are susceptible to EHV-1 infection, such as E. Derm cells and equine PBMC. Expression of MHC class I on E. Derm cells and PBMC was confirmed by flow cytometry using the same anti-MHC class I antibodies used in Fig. 2B,C (Fig. S1 in Supporting Information). After preincubation with these antibodies or the IgG isotype controls, cells were exposed to Ab4-GFP. PT85A and H58A treatment significantly inhibited virus entry into E. Derm cells (inhibition of 58% and 30% compared to the mock treatment, respectively) (Fig. 5A). PT85A and H58A also prevented EHV-1 entry into PBMC (infection inhibition of 33% and 65%, respectively) (Fig. 5B). On the other hand, treatment with B5C did not lead to inhibition of EHV-1 entry into these cells. These results suggest that equine MHC class I molecules are involved in EHV-1 entry into different types of equine cells.

### MHC class I molecules play a pivotal role in EHV-1 entry into E. Derm cells

**Figure 6:**
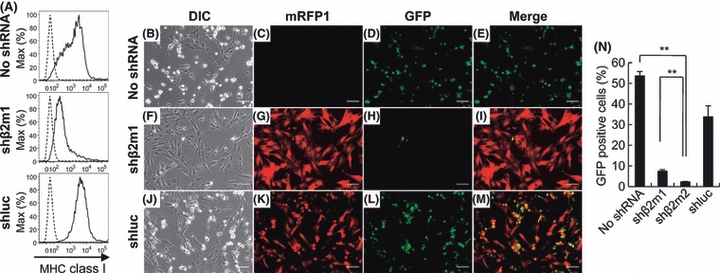
Knock-down of the cell surface expression of major histocompatibility complex (MHC) class I. E. Derm cells were infected with recombinant lentiviruses carrying both shRNA and mRFP1 expression cassettes. E. Derm cells were transduced with equine β2m-specific shRNA (shβ2m1 and shβ2m2) or luciferase-specific shRNA (shluc). (A) Flow cytometric analysis of the cell surface expression of MHC class I. Cells transduced with shRNAs (solid lines) or nontreated E. Derm cells (dashed line) were stained with anti-MHC class I antibody PT85A. (B–M) Effect of shRNA transduction on the susceptibility of E. Derm cells to equine herpesvirus-1 (EHV-1). Cells were infected with Ab4-GFP at an MOI of 1 for 24 h. Transduction of shRNA lentiviral vectors was confirmed by mRFP1 expression and infection of Ab4-GFP was confirmed by green fluorescent protein (GFP) expression under a fluorescence microscope. Scale bars: 100 μm. (N) After infection with Ab4-GFP, GFP-positive cells were counted by flow cytometry. The bars represent the means from three samples, and error bars show standard deviations. Statistical significance was analyzed by Student's *t*-test and is indicated by an asterisk (**P < 0.01).

As PT85A markedly prevented EHV-1 entry into E. Derm cells (Fig. 5A), we postulated that EHV-1 enters equine cells mainly via a MHC class I-dependent pathway. To test this hypothesis, we examined the effect of endogenous MHC class I gene knockdown on EHV-1 infection of E. Derm cells using lentiviral delivery of short hairpin RNAs (shRNAs). The recombinant lentivirus would drive shRNA synthesis under the control of the H1 promoter and express monomeric red fluorescent protein 1 (mRFP1) under the control of the cytomegalovirus promoter, thereby enabling us to identify lentivirus infected cells by mRFP1 gene expression (Fig. 6G,K). A transduction efficiency higher than 95% was estimated from mRFP1 expression.

We constructed recombinant viruses carrying shRNA that targeted equine MHC class I heavy chain mRNA. However, MHC class I expression was not sufficiently reduced (data not shown), probably due to MHC class I gene polymorphisms (Figueiredo *et al.* 2006).

MHC class I molecules are composed of a single membrane-spanning heavy chain paired with a soluble protein β2-microglobulin (β2m). The cell surface expression of MHC class I depends on the noncovalent binding of the heavy chain and β2m (Bjorkman & Parham 1990). Figueiredo *et al.* (2006) found that β2m-specific shRNA silenced MHC class I molecules on the cell surface. Therefore, we designed two shRNA constructs targeting equine β2m (designated shβ2m1 and shβ2m2). Flow cytometric analysis indicated that E. Derm cells transduced with β2m-specific shRNAs showed silenced β2m expression and reduced MHC class I cell surface expression compared to nontreated cells or cells transduced with luciferase-specific shRNA (shluc) (Fig. 6A).

Nontreated or shRNA-transduced E. Derm cells were then infected with Ab4-GFP. Although there were a few GFP-positive cells, almost all of the β2m-specific shRNA-transduced E. Derm cells showed neither CPE (Fig. 6F,G) nor GFP expression (Fig. 6H). In contrast, nontreated (Fig. 6B–E) and shluc-transduced (Fig. 6J–M) cells demonstrated both CPE and GFP expression after EHV-1 infection, indicating that these cells were still susceptible to EHV-1 infection. The number of EHV-1 infected cells was reduced in shβ2m1- and shβ2m2-transduced E. Derm cells when compared to mock cells (Fig. 6N). These results suggest that MHC class I is a major determinant of susceptibility to EHV-1 infection in E. Derm cells.

### MHC class I expression on CHO-K1 cells has no role in EHV-1 infection

**Figure 7:**
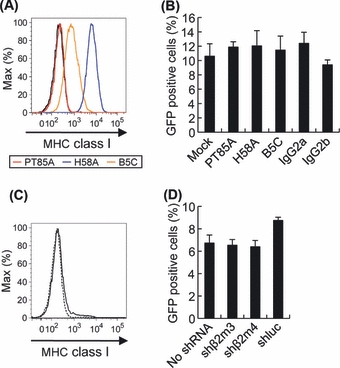
Major histocompatibility complex (MHC) class I-independent infection of CHO-K1 cells by equine herpesvirus-1 (EHV-1). (A) Flow cytometric detection of hamster MHC class I expression on CHO-K1 cells stained with anti-MHC class I antibody PT85A (red), H58A (blue), B5C (orange) or isotype controls IgG2a and IgG2b (black and gray, respectively). (B) CHO-K1 cells were preincubated with 50 μg/mL of anti-MHC class I or control antibodies for 30 min and then infected with the EHV-1 Ab4-GFP at an MOI of 5 in the presence of antibody for 2 h at 37 °C. After removing virus–antibody mixtures, cells were treated with a low-pH citrate buffer to inactivate extracellular virions. Incubation was continued for a further 12 h. Green fluorescent protein (GFP)-positive cells were counted by flow cytometry. Bars represent means from three samples, and error bars show standard deviations. (C, D) Knock-down of the cell surface expression of MHC class I by transduction of Chinese hamster's β2m-specific shRNA (shβ2m3 and shβ2m4). (C) Flow cytometric analysis of the cell surface expression of MHC class I. Cells expressing shβ2m4 (solid line) and no shRNA (dashed line) were stained with anti-MHC class I antibody H58A. (D) Cells expressing shRNA were infected with the EHV-1 Ab4-GFP at an MOI of 1 for 24 h. GFP-positive cells were counted by flow cytometry. Bars represent means from three samples, and error bars show standard deviations.

EHV-1 is able to infect CHO-K1 cells, which are naturally resistant to HSV-1, HSV-2, PRV and VZV entry. Therefore, we investigated whether MHC class I is also involved in EHV-1 entry into CHO-K1 cells. Anti-MHC class I antibody H58A and B5C reacted with MHC class I on CHO-K1 (Fig. 7A); however, these antibodies failed to block EHV-1 entry (Fig. 7B). PT85A did not recognize Chinese hamster's MHC class I (Fig. 7A). We also confirmed the influence of β2m knockdown on CHO-K1 cells. The surface expression of MHC class I was strongly reduced by the transduction of shRNAs targeting Chinese hamster's β2m (Fig. 7C). The levels of EHV-1 infection in β2m-knockdown cells were comparable to those in nontreated and shluc-transduced cells (Fig. 7D). These data indicate that EHV-1 does not depend on MHC class I for entry into CHO-K1 cells.

## Discussion

In this study, the surface expression of equine MHC class I heavy chains cloned from EBMECs or E. Derm cells rendered mouse NIH3T3 cells susceptible to EHV-1 infection, with EHV-1 entry and subsequent replication in these cells depending on the expression of MHC class I. Pretreatment of equine cells, including primary cultured EBMECs and PBMC which are important targets of EHV-1 *in vivo*, with an anti-equine MHC class I monoclonal antibody inhibited EHV-1 entry. Moreover, the susceptibility of E. Derm cells to EHV-1 infection was markedly inhibited by β2m gene knockdown, which subsequently reduced the surface expression of all MHC class I molecules. These results suggest that equine MHC class I plays an important role in EHV-1 entry into equine cells.

A mechanism of entry for EHV-1 was recently reported by another group (Kurtz *et al.* 2010); however, they demonstrated the involvement of MHC class I only in a case of EHV-1 entry into a mouse melanoma cell line with exogenously overexpressed MHC class I. Therefore, our study is the first to show that equine MHC class I molecules actually mediate EHV-1 entry into equine cell types, which are naturally susceptible to EHV-1 infection.

Alphaherpesviruses entry into target cells occurs a cascade of direct interactions between viral glycoproteins and receptors (Spear 2004; Heldwein & Krummenacher 2008). Receptors for HSV-1 on the host cell surface bind to HSV-1 gD, triggering viral entry into cells (Whitbeck *et al.* 1997; Krummenacher *et al.* 1998; Shukla *et al.* 1999). Blocking the interaction between entry receptors and gD inhibits HSV-1 entry and infection (Whitbeck *et al.* 1997; Geraghty *et al.* 1998; Shukla *et al.* 1999). A previous study showing the involvement of MHC class I in EHV-1 entry did not identify the viral protein that acts as a ligand for MHC class I (Kurtz *et al.* 2010). In this study, we demonstrated that EHV-1 gD interacts with equine MHC class I and that viral entry into 3T3-A68 cells was inhibited by treatment with anti-gD antibody and a soluble form of gD. These results suggest that equine MHC class I acts as an entry receptor for EHV-1 by binding to EHV-1 gD.

Our data showed that only a fraction of NIH3T3 cells expressing MHC class I was infected with EHV-1. This finding suggests that the cell surface expression of MHC class I is not the sole determinant for the susceptibility of NIH3T3 cells to EHV-1. Cellular factors other than equine MHC class I might be required for establishing highly efficient infection of NIH3T3 cells with EHV-1.

In this study, anti-MHC class I antibodies showed variable inhibition effects on EHV-1 infection in different cell types (Figs 2B,C, 5A,B). One possible explanation for this variability is that the extent of inhibition of EHV-1 entry by anti-MHC class I antibodies may be influenced by the number and/or diversity of MHC class I molecules expressed on each cell type. As MHC molecules are polymorphic, the degree of monoclonal antibody reactivity may vary among MHC class I molecules encoded by different loci.

Equine MHC class I genes are located on chromosome 20 (Ansari *et al.* 1988; Gustafson *et al.* 2003). It is still unclear how many class I genes exist within the horse genome, but the presence of up to 33 MHC class I genes and/or pseudo genes is indicated by restriction fragment length polymorphisms of the equine genome (Alexander *et al.* 1987). Moreover, 15 MHC class I loci were identified and sequenced from bacterial artificial chromosome clones containing homozygous MHC horse genes, and seven of these loci were transcribed into the mRNA in adult lymphocytes by RT-PCR (Tallmadge *et al.* 2005). Although our study showed that at least two equine MHC class I heavy chains, A68 and B118, confer susceptibility to EHV-1 infection, further studies are needed to determine whether all equine MHC class I molecules are involved in EHV-1 entry.

Although this study demonstrates the role of equine MHC class I in EHV-1 entry into equine cells, the distribution of MHC class I itself may not correlate directly with EHV-1 tissue tropism, as virtually all cell types can express MHC class I molecules (David-Watine *et al.* 1990). Therefore, host factors other than MHC class I may be involved in the observed EHV-1 tropism.

Recently, αV integrins were shown to be important for EHV-1 entry into CHO-K1 cells but not endothelial cells collected from the carotid arteries of horses, suggesting the existence of different pathways of EHV-1 entry in these cells (Van de Walle *et al.* 2008). Here, we demonstrated that MHC class I participates in EHV-1 entry into equine cells; however, pretreatment with anti-MHC class I antibody and the reduction of cell surface MHC class I expression by β2m knockdown did not alter the susceptibility of CHO-K1 cells to EHV-1. These data indicate that EHV-1 relies on different pathways for entry into different types of host cells.

The mouse intranasal infection model has many similarities to natural infection in horses. Intranasal infection of adult mice causes local viral replication in the respiratory mucosa, with formation of intranuclear inclusions and subsequent rhinopneumonitis (Awan *et al.* 1990; Inazu *et al.* 1993; Bartels *et al.* 1998). However, this model does not develop encephalomyelitis, a characteristic of viral infection in CNS endothelial cells. Our results suggest that EHV-1 is unlikely to utilize mouse MHC class I as a factor for entry into mouse cells because the NIH3T3 cells used in this study (which are not naturally susceptible to EHV-1 infection) expressed mouse MHC class I molecules (haplotype H2^q^) on their surface (Seliger *et al.* 1998; Herrmann *et al.* 2004). Interestingly, in contrast to the EBMECs that expressed equine MHC class I and were thus susceptible to EHV-1 infection, mouse brain microvascular endothelial cells are completely resistant to EHV-1 infection and are not susceptible to viral entry (Hasebe *et al.* 2006). Therefore, the differences between the host factors underlying EHV-1 entry in equine and mouse cells may contribute to the difference in EHV-1 pathogenicity between the horse and the mouse model.

In summary, we demonstrated that equine MHC class I molecule acts as a functional gD receptor for EHV-1 entry and infection, which contributes to the understanding of the molecular mechanisms of EHV-1 entry into cells (Table S1 in Supporting Information). In recent years, outbreaks of the neurologic EHV-1 have occurred at various equine facilities worldwide. An effective approach to therapy and prevention is still under investigation. Transgenic mice expressing equine MHC class I on their endothelial cells, already under development, might provide a suitable model for the study of EHV-1 pathogenesis, aiding in the investigation of novel vaccines and drugs for EHV-1 infection.

## Experimental procedures

### Cells and viruses

NIH3T3 and 293T cells were cultured in Dulbecco's modified Eagle medium (DMEM) containing 10% fetal bovine serum (FBS). E. Derm cells (CCL-57; ATCC, Manassas, VA, USA) were cultured in DMEM supplemented with 10% FBS and 0.1 mm nonessential amino acids. RK13 cells were cultured in Eagle's minimum essential medium (MEM) containing 10% FBS. CHO-K1 cells were cultured in DMEM/F-12 containing 10% FBS. PBMC were isolated from equine blood by density gradient centrifugation over Histopaque 1077 (Sigma, St Louis, MO, USA) and maintained in RPMI 1640 medium supplemented with 10% FBS, 50 μm 2-mercaptoethanol and 10 μg/mL gentamicin. Primary EBMECs were isolated from a horse brain as described previously (Hasebe *et al.* 2006) and were cultured in Medium 199 Earl's (Invitrogen, Carlsbad, CA, USA) supplemented with 10% FBS and 10 μg/mL gentamicin. All cells were maintained at 37 °C in 5% CO_2_. The EHV-1 mutant Ab4-GFP contains a GFP expression cassette between ORF 62 and ORF 63 (Ibrahim el *et al.* 2004). Stock viruses were cultured in E. Derm cells and titrated by plaque formation assay on RK13 cells.

### Plasmids

The pIRES2-DsRed-Express2 (Clontech Laboratories, Palo Alto, CA, USA) is a bicistronic expression vector containing the internal ribosome entry site 2 (IRES2) between the multiple cloning site and the red fluorescent marker protein DsRed-Express2 coding region. The pCXSN vector was generated by removing the c-myc epitope tag from pCMV-Myc (Clontech Laboratories) and replacing the multiple cloning site with *Xho* I, *Sal* I and *Not* I. The self-inactivating (SIN) lentiviral vector constructs (CSII-CMV-MCS-IRES2-Bsd and CS-RfA-CMV-mRFP1), entry vector (pENTR4-H1), packaging construct (pCAG-HIVgp) and the VSV-G- and Rev-expressing construct (pCMV-VSV-G-RSV-Rev) were kindly provided by Dr Miyoshi (RIKEN BioResource Center, Ibaraki, Japan). To generate the Ig fusion proteins, a pME18S expression vector containing a mouse CD150 leader segment at the N terminus and Fc segment of human IgG1 at C terminus (Satoh *et al.* 2008) was kindly provided by Dr Arase (Osaka University, Osaka, Japan). To construct the expression plasmids, cDNA fragments of equine MHC class I B118 and β2m and of H2K and H2D were respectively amplified from the total RNA of E. Derm cells and of C57BL/6 mice spleen by RT-PCR. cDNA fragments of EHV-1 gD were amplified from the genome of EHV-1 Ab4-GFP virus by PCR. A68 expression vectors containing HA epitope tag, pCXSN-A68-HA, were constructed using pCXSN-A68 as templates. PCR primers used in this study are listed in Table S2 in Supporting Information.

### Complementary DNA isolation

A unidirectional EBMECs cDNA library cloned into the *Eco* RI-*Xho* I site of the pcDNA 3.1 (+) vector (Invitrogen) in *Escherichia coli* DH10B was plated onto two hundred 150-mm LB agar plates containing ampicillin (100 μg/mL). Colonies were pooled by scraping and frozen in glycerol. Samples of each stock were combined into groups of 20 and cultured in LB broth with 100 μg/mL ampicillin at 37 °C. Plasmid DNA was prepared using a QIAprep Spin Miniprep kit (Qiagen, Valencia, CA, USA). NIH3T3 cells plated in six-well plates were transfected with plasmid DNA from each pool by using Lipofectamine 2000 (Invitrogen). Cells transfected with the GFP-expressing plasmid pEGFP-N1 (Clontech Laboratories) or with pcDNA 3.1 (+) were used as controls. Thirty hours after transfection, cells were infected with Ab4-GFP at a multiplicity of infection (MOI) of 5. At 24 h p.i., we determined the susceptibility of cells to Ab4-GFP by counting the number of GFP-expressing cells under an inverted fluorescence microscope (IX70; Olympus, Tokyo, Japan). The stock that yielded the largest number of infected cells was divided into one hundred pools. This process of subdivision and screening was repeated to obtain a single clone that yielded a plasmid with the desired phenotype.

### Establishment of an equine MHC class I-expressing cell line

To obtain a cell line stably expressing the equine MHC class I heavy chain gene, we used an HIV-1-based lentiviral vector pseudotyped with the vesicular stomatitis virus G glycoprotein (VSV-G) (Miyoshi *et al.* 1998). Briefly, an MHC class I heavy chain gene was cloned into the SIN lentiviral vector construct, CSII-CMV-MCS-IRES2-Bsd. A recombinant lentivirus vector was generated by transient transfection of 293T cells with a combination of the SIN lentiviral vector construct, pCAG-HIVgp and pCMV-VSV-G-RSV-Rev. The supernatant containing lentivirus vector was collected after incubation of the cells at 37 °C for 48 h. The A68 or B118-expressing NIH3T3 cell lines (3T3-A68 and 3T3-B118) were established by infecting NIH3T3 cells with each lentiviral vector and cultured in the presence of 10 μg/mL blasticidin S HCl (Invitrogen).

### Flow cytometry

Cells were detached with cell dissociation buffer (Invitrogen) and washed with phosphate-buffered saline (PBS) containing 2% bovine serum albumin. Cells were stained with the anti-MHC class I monoclonal antibody PT85A, H58A, B5C (VMRD, Pullman, WA, USA) or anti-gD polyclonal antibody (generated in our laboratory) at 4 °C for 30 min. Mouse IgG2a (BD Biosciences, San Jose, CA, USA), IgG2b and rabbit IgG (Beckman Coulter, Fullerton, CA, USA) were used as controls. Cells were stained with an Alexa Fluor 488-conjugated anti-mouse IgG antibody (Invitrogen), allophycocyanin (APC)-conjugated anti-mouse IgG or APC-conjugated anti-rabbit IgG (Beckman Coulter) at 4 °C for 30 min. Flow cytometric analysis was performed using a FACS Canto system (BD Biosciences), and the data collected were analyzed using Flowjo software (Tree Star, Ashland, OR, USA).

### Generation of Ig fusion protein

The expression vectors pME18S-A68-IgG Fc and pME18S-gD-IgG Fc were constructed by subcloning the extracellular domain of gD and A68 into pME18S expression vectors. Ig fusion proteins were expressed using the FreeStyle 293 expression system (Invitrogen) as described by the manufacturer. Briefly, 293F cells were transfected with pME18S-A68-IgG Fc and pCXSN-equine β2m (for A68-Ig expression) or pME18S-gD-IgG Fc (for gD-Ig expression) using 293fectin (Invitrogen). Secreted Ig fusion proteins were purified on HiTrap rProtein A FF column (GE Healthcare, Buckinghamshire, UK) and buffer exchanged into PBS using an Amicon Ultra-15 centrifugal filter unit with an Ultracel-10 membrane (Millipore, Bedford, MA, USA).

### Immunoprecipitation assay

293T cells were transfected with gD expression plasmid, or with HA-tagged A68 and equine β2m expression plasmids, to obtain a high expression of A68 using 293fectin. Cells were lysed in a lysis buffer [10 mm Tris–HCl (pH 7.5), 0.5% Brij 98, 150 mm NaCl] supplemented with complete protease inhibitor cocktail (Roche, Basel, Switzerland). Lysed proteins were incubated with Dynabeads Protein A (Invitrogen) for 1 h at 4 °C after coating with Ig fusion protein. Precipitated protein complexes were eluted with 0.1 m citrate buffer (pH 3.0) and fractionated by SDS-PAGE. Proteins were transferred onto Immobilon-P transfer membranes (Millipore) and detected with anti-gD polyclonal antibody, anti-HA monoclonal antibody (Sigma) or anti-human IgG antibody (Jackson Immunoresearch, West Grove, PA, USA).

### Binding assay

To detect virions attached to the cell surface, cells were detached and incubated with Ab4-GFP at an MOI of 20. After incubation for 1 h at 4 °C, unbound virus was washed out with PBS, stained with an anti-EHV-1 polyclonal antibody (a gift from Dr Kirisawa, Rakuno Gakuen University, Hokkaido, Japan) and analyzed by flow cytometry.

To assess Ig fusion protein binding, cells were detached and incubated with 5 μg of Ig fusion protein at 4 °C for 30 min. Purified human IgG (Invitrogen) was used as a control Ig protein. Binding of the Ig fusion proteins was detected by phycoerythrin (PE)-conjugated anti-human IgG antibody (Beckman Coulter) and analyzed by flow cytometry.

### ATP depletion assay

For samples depleted of cellular ATP during viral entry, cells were preincubated in ATP depletion media composed of glucose-free, FBS-free DMEM (Invitrogen) with 10 mm 2-deoxyglucose (Sigma) and 10 mm sodium azide (Sigma) for 30 min, and infected with Ab4-GFP at an MOI of 5 (RK13 and E. Derm) or 20 (3T3-A68 and CHO-K1) for 1 h in ATP depletion media. After EHV-1 infection, cells were treated with 0.1 m citrate buffer (pH 3.0) to inactivate remaining viruses on the cell surface. The media were replaced with regular culture media and cells were cultured for 12 h at 37 °C. For samples depleted of ATP after postentry, cells were infected with Ab4-GFP in FBS-free DMEM. After viral infection, cells were incubated in ATP depletion media for 1.5 h, and then the media were replaced with regular culture media. At 12 h p.i., GFP-positive cells were counted by flow cytometry. Cellular ATP levels of each cell line treated or untreated with the 2-deoxyglucose and sodium azide were measured using the CellTiter-Glo luminescent cell viability assay (Promega, Madison, WI, USA) according to the manufacturer's instructions.

### Detection of immediate early, early and late EHV-1 mRNA expression

Cells were infected with Ab4-GFP at an MOI of 5. After washing cells with PBS, total RNA was extracted at 0 h p.i. (immediately after seeding the virus) and 4 h p.i. with TRIzol reagent (Invitrogen). After treatment with amplification-grade DNase I (Invitrogen), RT-PCR was performed with primers specific for EHV-1 immediate early (IE), early (ICP0) and late (gB, gD, gK) genes, using the method described by Hasebe *et al.* (2006).

### Viral growth analysis

Cells were infected with Ab4-GFP at an MOI of 5. After incubation at 37 °C for 1 h to allow viral attachment, cells were washed three times with PBS and re-fed with growth media. At 1 h p.i. (immediately after the PBS wash), 8 and 24 h p.i., the supernatants were collected. The viral titer was determined by plaque formation on RK13 cells.

### Infectivity neutralization assay

For neutralization of viral gD, EHV-1 Ab4-GFP at an MOI of 5 were incubated with anti-gD polyclonal antibody or control rabbit IgG for 30 min at 37 °C, and the virus–antibody mixture was added to the cells. After incubation for 2 h, extracellular virus was inactivated by treatment with 0.1 m citrate buffer (pH 3.0). The cells were maintained in fresh growth medium for further 12 h p.i. Viral entry was assessed by counting the number of GFP-positive cells using flow cytometry.

For competition of viral gD, 3T3-A68 cells were incubated with gD-Ig fusion protein for 30 min at 37 °C, followed by infected 3T3-A68 cells for 2 h. Viral entry was evaluated as described above.

For examination of the effects of anti-MHC class I antibodies, cells were incubated with 50 μg/mL of antibodies for 30 min at 37 °C. The cells were infected with Ab4-GFP virus at an MOI of 5 for 2 h at 37 °C. Viral entry was evaluated as described above.

### Lentiviral vector expressing shRNA

Lentiviruses carrying shRNA were generated using the method described by Katayama *et al.* (2004). We designed shRNA constructs, shβ2m1 and shβ2m2, that were specific for the equine β2m sequence (GenBank accession number: X69083), and shβ2m3 and shβ2m4 that were specific for Chinese hamster β2m sequence (GenBank accession number: X57112). We also generated shRNAs against luciferase (shluc) as a control (Ui-Tei *et al.* 2004). Briefly, the entry vectors pENTR4-H1-shRNA were constructed by inverse PCR using the pENTR4-H1 and synthetic primers (listed in Table S2 in Supporting Information). Each entry vector was incubated with the CS-RfA-CMV-mRFP1 vector in the presence of Gateway LR Clonase (Invitrogen) for plasmid recombination. The Clonase-recombinant SIN vector constructs, CS-H1-shRNA-mRFP1, were transfected into 293T cells with the Lenti-X HT packaging system (Clontech Laboratories).

### β2-Microglobulin knockdown

Supernatants containing each lentivirus were applied to E. Derm or CHO-K1 cells. Transduction of shRNA into cells was confirmed by mRFP1 expression. The effect of shRNA knockdown was evaluated from the decreased level of MHC class I heavy chain expression using flow cytometry. Cells expressing shRNA were infected with Ab4-GFP at an MOI of 1 for 24 h. Fluorescence signals of mRFP1 and GFP were observed under an inverted fluorescence microscope, and images were processed using DP manager software (Olympus). GFP-positive cells were counted by flow cytometry.

### Statistical analysis

Statistically significant differences were determined by Student's *t*-tests. Results are presented as arithmetic means, and bars represent standard deviations.

### Accession numbers

The reported nucleotide sequence data are available in the DDBJ/EMBL/GenBank databases under accession numbers AB525079 (A68) and AB525080 (B118).

## Supporting Information/Supplementary material

The following Supporting Information can be found in the online version of the article:

**Figure S1** Flow cytometric detection of MHC class I expression on E. Derm, EBMECs and PBMC cells stained with anti-MHC class I antibody PT85A, H58A, B5C or isotype controls IgG2a and IgG2b.

**Figure S2** Cellular ATP levels of each cell line with or without the ATP depletion treatment.

**Table S1** Cellular entry pathways of EHV-1

**Table S2** List of primers used to construct vectors
