# Supplementary material for: Equine major histocompatibility complex class I molecules act as entry receptors that bind to equine herpesvirus-1 glycoprotein D
Source: Genes Cells. 2011 Apr;16(4):343–57. doi: 10.1111/j.1365-2443.2011.01491.x (PMC3118799; doi:10.1111/j.1365-2443.2011.01491.x)
Supplement: Supplementary file 1 [file gtc0016-0343-SD1.pdf]

FIGURE LEGENDS OF SUPPORTING INFORMATION

Figure S1. Flow cytometric detection of MHC class I expression on E. Derm, EBMECs and PBMC cells stained with anti-MHC class I antibody PT85A (red), H58A (blue), B5C (orange), or isotype controls IgG2a and IgG2b (black and gray, respectively).

Figure S2. Cellular ATP levels of each cell line with or without the ATP depletion treatment. RK13, 3T3-A68, CHO-K1 and E. Derm cells were incubated with ATP depletion media (open circles) or DMEM control media (solid squares) for the indicated time periods. Cellular ATP was measured with the CellTiter-Glo luminescent cell viability assay. Error bars represent standard deviations.

Fig. S1

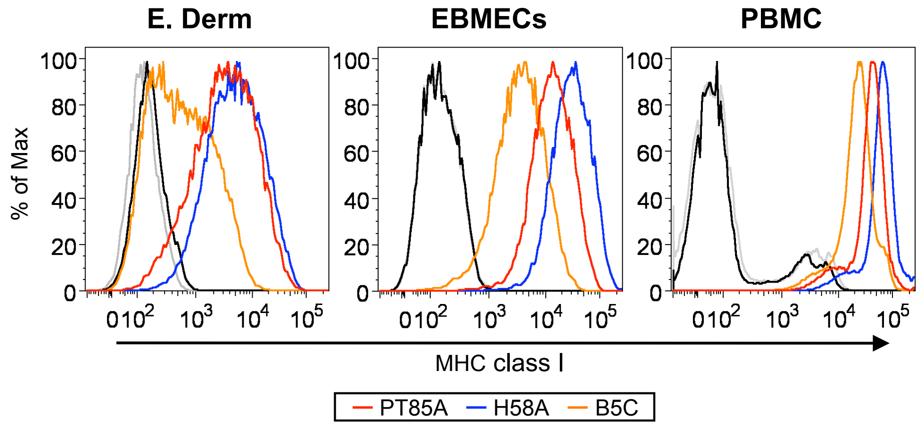
E. Derm
EBMECs
PBMC
% of Max
100
80
60
40
20
0
0 10^2 10^3 10^4 10^5
MHC class I
PT85A
H58A
B5C

Fig. S2

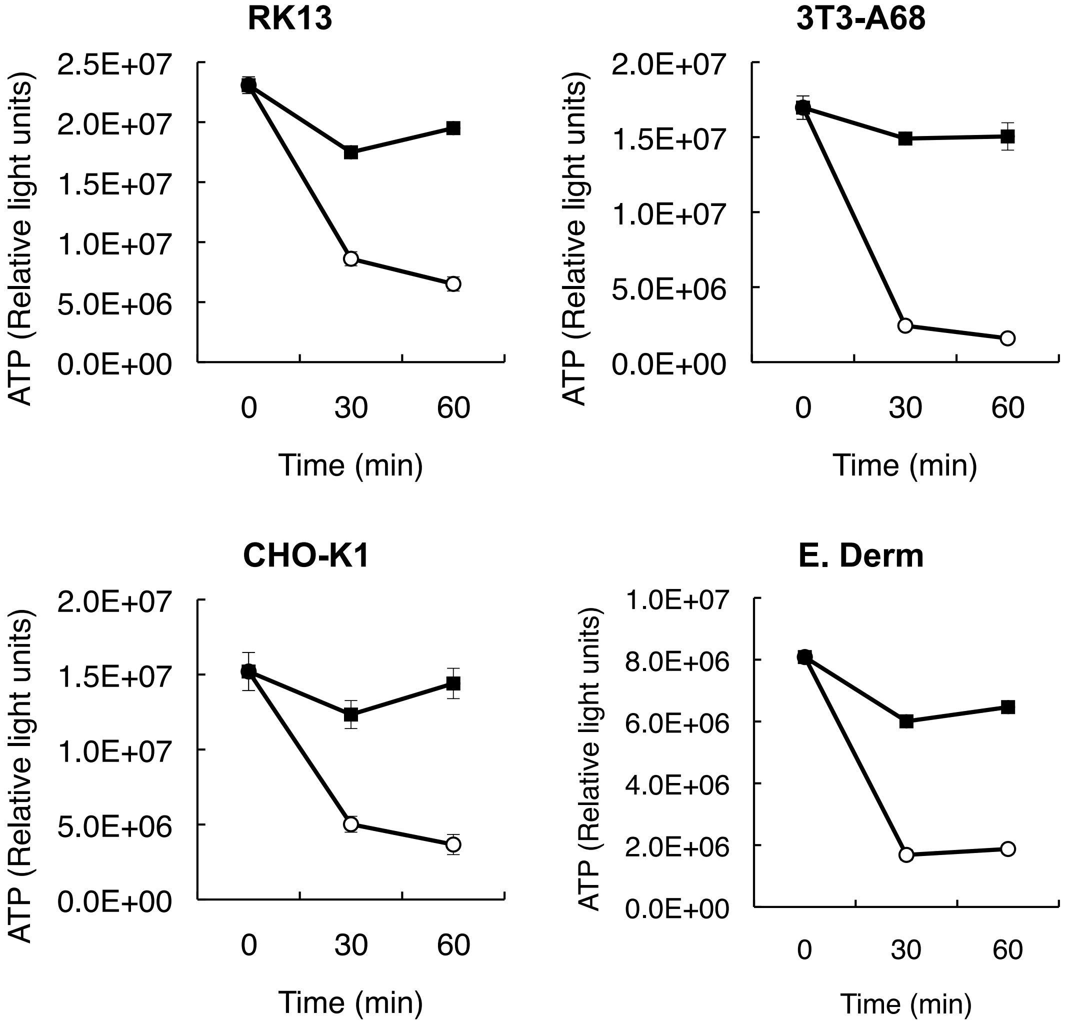
RK13
ATP (Relative light units)
2.5E+07
2.0E+07
1.5E+07
1.0E+07
5.0E+06
0.0E+00
0
30
60
Time (min)
3T3-A68
ATP (Relative light units)
2.0E+07
1.5E+07
1.0E+07
5.0E+06
0.0E+00
0
30
60
Time (min)
CHO-K1
ATP (Relative light units)
2.0E+07
1.5E+07
1.0E+07
5.0E+06
0.0E+00
0
30
60
Time (min)
E. Derm
ATP (Relative light units)
1.0E+07
8.0E+06
6.0E+06
4.0E+06
2.0E+06
0.0E+00
0
30
60
Time (min)
