# Supplementary material for: Equine major histocompatibility complex class I molecules act as entry receptors that bind to equine herpesvirus-1 glycoprotein D
Source: Genes Cells. 2011 Apr;16(4):343–57. doi: 10.1111/j.1365-2443.2011.01491.x (PMC3118799; doi:10.1111/j.1365-2443.2011.01491.x)
Supplement: Supplementary file 2 [file gtc0016-0343-SD2.pdf]

Table S1 Cellular entry pathways of EHV-1

Cell types	Species	Entry pathways	Receptor molecules	References
Equine aortic endothelial cells	Horse	Direct fusion	Unclear	Van de Walle et al. (2009)
Equine brain microvascular endothelial cells	Horse	Endocytosis	MHC class I	Hasebe et al. (2006), Hasebe et al. (2009), Current study
Equine peripheral blood mononuclear cells	Horse	Endocytosis	MHC class I and Integrin	Van de Walle et al. (2009), Current study
E. Derm cells	Horse	Direct fusion and Endocytosis	MHC class I	Frampton et al. (2007), Hasebe et al. (2009), Current study
CHO-K1 cells	Hamster	Endocytosis	Integrin	Frampton et al. (2007), Van de Walle et al. (2009)
RK13 cells	Rabbit	Direct fusion	Unclear	Frampton et al. (2007)
