# Supplementary material for: Equine major histocompatibility complex class I molecules act as entry receptors that bind to equine herpesvirus-1 glycoprotein D
Source: Genes Cells. 2011 Apr;16(4):343–57. doi: 10.1111/j.1365-2443.2011.01491.x (PMC3118799; doi:10.1111/j.1365-2443.2011.01491.x)
Supplement: Supplementary file 3 [file gtc0016-0343-SD3.pdf]

Table S2 List of primers used to construct vectors

Construct name	Used primer pair	
pA68-DsRed	forward	5'-CCCTCGAGACCATGTGGGTCATGGAGCCTCG-3'
	reverse	5'-TGGAATTCTTACACTTTAGGATCCGTGAGAG-3'
pB118-DsRed	forward	5'-CCCTCGAGACCATGATGCCCCCAACCTTCCTC-3'
	reverse	5'-AGGAATTCTCACACTTTCTGTGGGAGAGA-3'
pluc-DsRed	forward	5'-AACCTCGAGATGGAAGACGCCAAAAACAT-3'
	reverse	5'-AGGAATTCTTAGAGGTCTCGATCCCACTT-3'
pCXSN-A68	forward	5'-CCCTCGAGACCATGTGGGTCATGGAGCCTCG-3'
	reverse	5'-AAGCGGCCGCTTACACTTTAGGATCCGTGAGAG-3'
pCXSN-A68-HA	forward	5'-CCCTCGAGACCATGTGGGTCATGGAGCCTCG-3'
	reverse	5'-AAGCGGCCGCTTAAGCGTAATCTGGAACATCGTATGGGTACACTTTAGGATCCGTGAGAG-3'
pCXSN-equine β2m	forward	5'-CCCTCGAGACCATGGCTCGCGTCGTGGCGCT-3'
	reverse	5'-AAGCGGCCGCTCAGAGGTCTCGATCCCACTTAAC-3'
pCXSN-gD	forward	5`-CCCTCGAGACCATGTCTACCTTCAAGCTTAT-3`
	reverse	5`-AAGCGGCCGCTTACGGAAGCTGGGTATATTTAAC-3`
pME18S-A68-IgG Fc	forward	5`-CCCTCGAGGGCTCCCACTCCATGATGTA-3`
	reverse	5`-CCCTCGAGCCATCTCAGGGTGACGGGCTC-3`
pME18S-gD-IgG Fc	forward	5`-CCCTCGAGGCGGTTCGAGGACGCCAGG-3`
	reverse	5`-CCCTCGAGACCGACGCTGATGCCCACA-3`
pENTR-H1-shβ2m1	forward	5'-TCCGTTGGGTGACGTGAGTAAACCTTTTTGGAAATCTAGACCCAGCTTTCTTG-3'
	reverse	5'-CAGCACACGTTGAGTGACATGAGCAAACCGGGGATCTGTGGTCTCATACAGAAC-3'
pENTR-H1-shβ2m2	forward	5'-TCCGTTGGAACCCAGAGACATAGCTTTTTGGAAATCTAGACCCAGCTTTCTTG-3'
	reverse	5'-CAGCACACGTTGAAACCCAGAGACACAGCGGGGATCTGTGGTCTCATACAGAAC-3'
pENTR-H1-shβ2m3	forward	5'-TCCGTTGGCATCCAAGCAGACCACTTTTTGGAAATCTAGACCCAGCTTTCTTG-3'
	reverse	5'-CAGCACACGTTGACATCCAAGCAAACCACGGGGATCTGTGGTCTCATACAGAAC-3'
pENTR-H1-shβ2m4	forward	5'-TCCGTTGAAATTGAGACACATAGCTTTTTGGAAATCTAGACCCAGCTTTCTTG-3'
	reverse	5'-CAGCACACGTTGAAACTGAGACACACAGCGGGGATCTGTGGTCTCATACAGAAC-3'
pENTR-H1-shluc	forward	5'-TCCGTATTAAGACGACTCGAAATCTTTTTGGAAATCTAGACCCAGCTTTCTTG-3'
	reverse	5'-CAGCACACGTATCAAGACGACCCGAAATCGGGGATCTGTGGTCTCATACAGAAC-3'
